# Hypoglycaemia in Type 2 diabetes

**DOI:** 10.1111/j.1464-5491.2007.02341.x

**Published:** 2008-03-01

**Authors:** S A Amiel, T Dixon, R Mann, K Jameson

**Affiliations:** Kings College London School of Medicine London; * JB Medical Ltd Sudbury, UK; † School of Health and Related Research, University of Sheffield Sheffield, UK; ‡ Outcomes Research, Merck Sharp & Dohme Ltd Hoddesdon, UK

**Keywords:** burden of disease, consequences, hypoglycaemia, risk factors, Type 2 diabetes

## Abstract

The primary cause of hypoglycaemia in Type 2 diabetes is diabetes medication—in particular, those which raise insulin levels independently of blood glucose, such as sulphonylureas (SUs) and exogenous insulin. The risk of hypoglycaemia is increased in older patients, those with longer diabetes duration, lesser insulin reserve and perhaps in the drive for strict glycaemic control. Differing definitions, data collection methods, drug type/regimen and patient populations make comparing rates of hypoglycaemia difficult. It is clear that patients taking insulin have the highest rates of self-reported severe hypoglycaemia (25% in patients who have been taking insulin for > 5 years). SUs are associated with significantly lower rates of severe hypoglycaemia. However, large numbers of patients take SUs in the UK, and it is estimated that each year > 5000 patients will experience a severe event caused by their SU therapy which will require emergency intervention. Hypoglycaemia has substantial clinical impact, in terms of mortality, morbidity and quality of life. The cost implications of severe episodes—both direct hospital costs and indirect costs—are considerable: it is estimated that each hospital admission for severe hypoglycaemia costs around £1000. Hypoglycaemia and fear of hypoglycaemia limit the ability of current diabetes medications to achieve and maintain optimal levels of glycaemic control. Newer therapies, which focus on the incretin axis, may carry a lower risk of hypoglycaemia. Their use, and more prudent use of older therapies with low risk of hypoglycaemia, may help patients achieve improved glucose control for longer, and reduce the risk of diabetic complications.

Diabet. Med. 25, 245–254 (2008)

## Introduction

In healthy individuals, blood glucose concentrations are maintained within a very narrow range, despite major fluctuations in glucose entry into the body and glucose utilization in tissue metabolism. In people with diabetes mellitus, inadequate insulin secretion results in high blood glucose concentrations. The treatment of diabetes mellitus focuses on avoidance of hyperglycaemia in order to avoid its associated symptoms and to minimize the risk of vascular complications over time.

Treatments that elevate insulin concentrations in the blood independent of the ambient glucose inevitably carry risk of intermittent hypoglycaemia. Episodes of hypoglycaemia are distressing, either because of the symptom response to the falling blood glucose concentration, or because of the alteration in brain function that occurs if the plasma glucose falls too low to sustain normal neuronal function. Hypoglycaemia, particularly when severe, is associated with considerable cost, both to the individual and to the health service.

Most of the research into hypoglycaemia has used insulin-induced hypoglycaemia as a tool and has looked at hypoglycaemia in the insulin-deficient Type 1 diabetic population. The occurrence of hypoglycaemia in the treatment of Type 2 diabetes is also well recognized, but is more protean in nature, having different risk factors and clinical features according to the nature of the hypoglycaemic therapy, the extent of the insulin secretory deficit and the duration of diabetes. With the increasing drive for more strict glucose control in Type 2 diabetes and new therapies which may carry different risks for hypoglycaemia from established therapies, a review of what is known about hypoglycaemia in Type 2 diabetes is timely.

## Search methodology

A Medline search for relevant papers between 1966 and August 2006 was undertaken, using Web of Knowledge and PubMed. A broad search strategy was used and main search terms included: Type 2 diabetes, Type 2 diabetes mellitus, Type 2 diabetic; hypoglycemia, hypoglycaemia, hypoglycemic, hypoglycaemic; sulphonylurea, sulfonylurea, pioglitazone, rosiglitazone, metformin, insulin; prevalence, incidence, cost, fear of hypoglycemia/hypoglycaemia, health-related quality of life, HRQoL, health-related utility, satisfaction, compliance/adherence.

Main search terms were used in different combinations with the terms from the issues to be addressed. Search terms were truncated where necessary. In addition, we examined relevant United Kingdom Prospective Diabetes Study (UKPDS) papers, European Medicines Agency (EMEA), American Diabetes Association (ADA) and Canadian Diabetes Association (CDA) guidelines, references to papers of interest not identified via the electronic search but retrieved in the reference columns of those that were, and very recent references described at relevant meetings.

All papers concerning hypoglycaemia were considered eligible. However, only the most pertinent papers which addressed the issues of prevalence/incidence, cost of hypoglycaemia, fear of hypoglycaemia, health-related quality of life (HRQoL) associated with hypoglycaemia, satisfaction and compliance were included in the final review. This limitation was set to ensure the most relevant information to the topics were included.

Papers where the abstract had not been translated into English were excluded, as were papers which did not pertain to human adult populations with Type 2 diabetes. The initial number of ‘hits’ from the search was 1946 papers. Papers that were excluded totalled 1893 (not published in English, Type 1 diabetes, children, title of study deemed to be outside the scope or not relevant to the scope of the review, other). Fifty-three papers were therefore deemed eligible to best summarize or present evidence. Additional references relating to hypoglycaemia in patients with Type 2 diabetes were included if published after August 2006 or providing supplementary information.

## Definitions of hypoglycaemia

There is no consensus definition of hypoglycaemia in diabetes, and a variety of criteria have been used to define hypoglycaemic events. An early, very practical, definition of hypoglycaemia was the presence of Whipple's triad: decreased plasma glucose concentration, symptoms compatible with hypoglycaemia and rapid attenuation of those symptoms by correction of the low glucose. With the recognition of hypoglycaemia occurring without subjective awareness, this definition requires the addition of ‘and signs’ to the ‘symptoms’ of item two, but otherwise remains relevant to current practice. More recent definitions have been provided by the ADA [1], the CDA [2] and the EMEA [3]. These groups have attempted to define the clinical severity of hypoglycaemia, classify the event according to the presence or absence of a plasma glucose test and identify a threshold level for plasma glucose at which hypoglycaemia is diagnosed. Each group has defined a different level for this threshold, ranging from < 3.9 down to < 3.0 mmol/l. This lack of consensus makes it difficult to compare studies or quantify the frequency of hypoglycaemia in Type 2 diabetes [4].

For the proper interpretation of published studies, a discussion of the definition of the glucose threshold defining hypoglycaemia is necessary. For a biochemical definition, surgeons, defining spontaneous pathological hypoglycaemia requiring investigation and treatment, and forensic pathologists have used glucose concentrations of < 2.2 mmol/l, to avoid defining healthy people as hypoglycaemic [5,6]. At the other extreme, the recent ADA consensus [1] has defined any glucose concentration of < 3.9 mmol/l as hypoglycaemia, based on the reduction in endogenous insulin and increase in pancreatic glucagon which can be demonstrated at this level. However, plasma glucose can fall lower than this in health, especially in women. Furthermore, insulin-deficient patients with diabetes have lost the ability to modulate either insulin or glucagon in response to hypoglycaemia and depend instead on autonomic activation, subjective awareness and adrenaline to defend against severe hypoglycaemia [7]. Defining hypoglycaemia as any value < 3.9 mmol/l is likely to lead to overestimation of clinically significant hypoglycaemia associated with any specific diabetes therapy. The EMEA [3] recommend a value of < 3.0 mmol/l to define hypoglycaemia when assessing hypoglycaemic risk of different treatment regimens. This has the virtue of robustly detecting hypoglycaemia of clinical significance. Impaired cognitive function is seen at plasma glucose concentrations of < 3.0 mmol/l [8,9], and avoidance of plasma glucose concentrations of < 3.0 mmol/l has been able to restore hypoglycaemia awareness to people with Type 1 diabetes and defective counterregulation [10]. Avoidance of exposure to < 3.0 mmol/l is therefore clinically very desirable, whereas exposure to glucose concentrations of 3.5–4.0 mmol/l, provided the glucose fall is then arrested, is of little clinical significance. Because of the clear activation of counterregulatory responses at arterialized plasma glucose concentrations < 3.5 mmol/l, this intermediate number is also a reasonable definition of hypoglycaemia for use in clinical practice. Parenthetically, hypoglycaemia as an undesirable side effect of diabetes therapy should be distinguished from the glucose concentration set as the lower limit to a therapeutic target, which is correctly set higher (e.g. 4.0–4.5 mmol/l), within the physiological range.

There is a degree of consensus when defining hypoglycaemia by clinical picture alone. Most authorities [1–3] now accept that severe hypoglycaemia is an episode in which the mental state of a patient is so disturbed that they are unable to self-treat. The defining feature is the need for assistance from another person, with subdivisions for cases where parenteral therapy is required or coma or seizure occur. Mild hypoglycaemia encompasses all other episodes, recognized by the patient and self-treated. The category of moderate hypoglycaemia, self-treated episodes involving significant disruption to lifestyle, is now little used because of its imprecision and subjective nature. It is important to recognize that people with diabetes may refer to very symptomatic episodes of hypoglycaemia as severe when from a medical perspective they are justifiably mild, because the symptomatic patient self-treats and avoids the morbidity of impaired conscious level.

The lack of consensus regarding definitions makes it difficult to compare rates of hypoglycaemia across studies. Clinically this is important, since treatment decisions may be made on the evidence that one treatment is associated with less hypoglycaemia than another. For the benefit of changing therapies to be real, it is important to demonstrate that the prevented hypoglycaemic events would have been clinically problematic.

## Risk factors for hypoglycaemia in Type 2 diabetes

The most common cause of hypoglycaemia in Type 2 diabetes, resulting in significant physical and psychosocial morbidity, is iatrogenic, occurring with the use of insulin secretagogues and insulin therapy [11]. These can overwhelm the normal defences that should protect against a significant fall in plasma glucose concentration, primarily by preventing a compensatory fall in circulating insulin. Risk of severe hypoglycaemia is further increased by any defects in the other systems for maintaining glucose concentrations.

Defects in glucagon responses to hypoglycaemia develop in Type 2 diabetes [12], and some patients develop defects in the other stress responses. Specific therapies may worsen these defects; for example, sulphonylurea (SU) therapy sustains intra-pancreatic insulin levels during hypoglycaemia, which may further impair glucagon responses [13].

Risk factors for individual episodes of hypoglycaemia in patients with Type 2 diabetes include behavioural, physiological and therapeutic factors (see Table 1) [11,14–16]. The most common behavioural factor which precipitates individual episodes of severe hypoglycaemia that has been identified is missed or irregular meals [17,18]. Other lifestyle factors include alcohol, exercise and incorrect use of glucose-lowering medication (dose/timing) [11].

**Table 1:** Factors that may increase risk of hypoglycaemia during diabetes therapy

Impaired drug clearance
e.g. renal impairment, hepatic failure, hypothyroidism
Impaired counterregulatory capacity
e.g. Addison's disease, growth hormone deficiency, hypopituitarism
Increased peripheral glucose uptake
e.g. exercise
Decreased endogenous glucose production
e.g. liver failure, alcohol
Impaired glucose absorption
e.g. malabsorption, anorexia
Concurrent medications
Decreased renal excretion of SUs
e.g. aspirin, allopurinol
Displacement of SUs from albumin
e.g. aspirin, warfarin, sulphonamides, trimethoprim, fibrates
Decreased metabolism of SUs
e.g. warfarin, mono-amine oxidase inhibitors
Insulin secretagogue activity
e.g. non-steroidal anti-inflammatory agents

Therapeutic/physiological factors associated with increased risk include older age, duration of diabetes, presence of comorbidities, renal impairment, loss of residual insulin secretion, defective counterregulation and loss of awareness of hypoglycaemia [7,12,19–23]. Use of other medications can also increase risk. Some of these factors are interrelated, as increasing diabetes duration is inevitably associated with increasing age and increasing loss of endogenous insulin secretion.

Patient age also affects subjective awareness of hypoglycaemia. In the elderly, neuroglycopenic symptoms specifically related to articulation and coordination, which include unsteadiness, blurred or double vision, lack of coordination and slurred speech, are more common [24]. There are experimental data to show that symptoms of hypoglycaemia decline with increasing age, whereas the tendency for cognitive dysfunction in hypoglycaemia increases [25].

Time of day is also important—even in the absence of pharmacological therapy, the lowest plasma glucose of the day is just before the evening meal and unsuspected hypoglycaemia can occur at this time once drug therapy is started [26,27]. Intensification of treatment targets has also increased frequency of severe hypoglycaemia, although this effect will depend on the nature of the treatment used and the degree of insulin deficiency in the patient [21].

**FIGURE 1:**
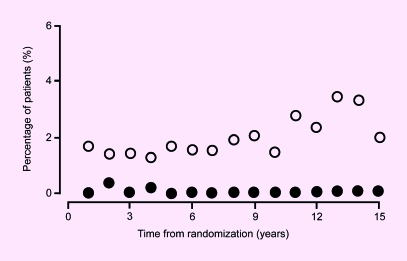
The effect of duration of diabetes on the proportion of patients experiencing severe hypoglycaemic episodes. The open circles represent patients in the intensive arm in the United Kingdom Prospective Diabetes Study (UKPDS) and show an increase in the proportion of patients experiencing a severe hypoglycaemic event over time. In contrast the closed circles represent patients in the diet-only arm of UKPDS and do not show an increase [21]. Reprinted from *The Lancet*, **352:** UKPDS 33. Intensive blood-glucose control with sulphonylureas or insulin compared with conventional treatment and risk of complications in patients with type 2 diabetes, 837–853, Copyright 1998, with permission from Elsevier.

Epidemiological data suggest that the incidence of hypoglycaemia in patients with Type 2 diabetes is highest when patients are using insulin [21]. However, this risk is strongly influenced by the clinical status of the patient. In the UKPDS, rates of severe hypoglycaemia rose once known diabetes duration exceeded 9 years (see Fig. 1).

## The size of the problem

Data on frequency of hypoglycaemia must be interpreted with caution for a number of reasons, including lack of consistency in definition, changes in conventions of care over time, and the duration of diabetes and degree of insulin deficiency. Data may be skewed, since some patients experience very little hypoglycaemia whereas others experience numerous episodes. Rates of events and numbers of patients affected may give different results.

Differences in the methods of data collection are also important. Although patient report is commonly used, inaccuracy of recall is well documented [28]. Using patient databases may underestimate the rate of hypoglycaemia, since few patients report hypoglycaemia to their doctor. In one study, only 15% of patients who experienced a mild/moderate event reported the incident to their doctor at the next scheduled visit. Only 2.5% of patients experiencing a severe episode reported additional visits to their general practitioner (GP) or hospital specialist after the episode [29]. On a speculative note, some patients may not admit hypoglycaemic events to physicians for fear of appearing unable to manage their condition.

### Insulin sensitizers (metformin and peroxisome proliferator-activated receptor gamma-agonists)

In patients on lifestyle adjustment and/or insulin sensitizing treatments, the risk for hypoglycaemia is negligible. UKPDS 73 [30] showed rates (based on patient report) of 0.1% and 0.3% for lifestyle and metformin, respectively, in patients receiving monotherapy or diet for 6 years from diagnosis. The recent ADOPT study has reported rates of around 10% on insulin sensitizers (metformin or rosiglitazone) over the 5 years of treatment, all self-reported [31]. Severe episodes were reported in very few patients (0.1%) on either treatment.

Patients are more at risk of hypoglycaemia when the insulin sensitizer is combined with insulin or insulin secretagogues. For example, the recent PROactive trial, comparing the addition of the peroxisome proliferator-activated receptor gamma (PPARγ)-agonist pioglitazone or placebo with usual diabetic treatment in Type 2 patients at high risk of vascular disease, found that the incidence of hypoglycaemia was significantly higher in the pioglitazone plus usual treatment group (28% vs. 20%, *P* < 0.001), although the incidence of severe hypoglycaemia (defined as hypoglycaemia which resulted in admission to hospital) was not significantly different (0.7% vs. 0.4%, *P* = 0.14). In the study, usual treatment included metformin, SU and insulin, either individually or in combinations and the data were not broken down by therapeutic agent [32]. Parenthetically, hypoglycaemia with α-glucosidase inhibitors is not a major risk [33].

### Sulphonylureas and insulin secretagogues

In the UKPDS, in the first year, 31% of patients treated with glibenclamide experienced mild hypoglycaemic symptoms [34]. Over the first 10 years, hypoglycaemia was reported by a mean of 18% of patients treated with glibenclamide per year [21].

A recent study commissioned by the Department for Transport used formalized self reporting and continuous glucose monitoring to compare rates (proportion of patients experiencing an event) of hypoglycaemia prospectively over 9–12 months between patients with Type 2 diabetes treated with SU, patients with Type 2 diabetes treated with insulin for < 2 years and those treated for > 5 years, as well as patients with Type 1 diabetes diagnosed within the last 5 years and those with long duration (> 15 years) of Type 1 diabetes (see next section for insulin data) [35]. Self-reported rates of mild and severe (any episode requiring third-party help) hypoglycaemia were 39% and 7%, respectively, in patients with Type 2 diabetes on SU. Rates of hypoglycaemia defined by values of < 2.2 mmol/l for at least 20 min on continuous glucose monitoring were 14% in patients with Type 2 diabetes on SUs [35].

Conflicting data exist with regard to severe hypoglycaemic events. Data derived from the Diabetes Audit and Research in Tayside Scotland/Medicines Monitoring Unit Collaboration (DARTS-MEMO) databases have revealed an annual rate of severe events [defined as requiring emergency treatment from Primary Care, ambulance, and Accident and Emergency (A&E) or hospital services, with blood glucose < 3.5 mmol/l associated with the need for treatment] of 0.8% (or 0.9 events per 100 patient-years) [22]. Older studies provide incidence rates of 0.75 per 100 person-years (short-acting agents up to 0.24 per 100 person-years) [36] and 1.23 per 100 person-years [19].

Data derived from the UK General Practice Research Database (719 GP practices, 34 052 patient-years of SU therapy) have reported that in users of SU, the annual risk of having one recorded diagnosis of any hypoglycaemic event is 1.8%, rising to 2.0% in those aged > 65 years. The risk of SU was greatest for glibenclamide: the study reported 25% fewer recorded episodes for gliclazide and 40% fewer for glipizide compared with glibenclamide [20]. These data should be interpreted with caution, since few patients report hypoglycaemia to their GP [29]. Recorded diagnoses were used to calculate rates of hypoglycaemia, and the study disregarded multiple diagnoses of hypoglycaemia in the notes. However, these potential quantitative problems should have existed equally for all therapies [20].

Hypoglycaemia rates with the third-generation SUs (e.g. glimepiride, glipizide and gliclazide) and the metiglinides (e.g. repaglinide and nateglinide) appear to be lower than those with glibenclamide and chlorpropamide. This is thought to be partly related to duration of action [36], but there may be other contributory factors. There is some evidence for differential effects on insulin sensitivity, for example [37].

A prospective population-based study carried out in Germany has found that rates of severe hypoglycaemia (defined as a requirement for glucose or glucagon injection and blood glucose < 2.8 mmol/l) with glimepiride were significantly lower than with glibenclamide at 0.86 per 1000 person-years vs. 5.6 per 1000 person-years [38]. Some severe hypoglycaemia occurred with both agents, and the patients involved were elderly (mean age 84 years), had very tight glycaemic control and > 60% had impaired renal function, irrespective of drug used. A later study of episodes of severe hypoglycaemia on either drug has failed to show any differences between the clinical course and revealed that severe events were present at all doses in both agents [39].

Pooled data from double-blind active-comparator studies comparing repaglinide with SU have shown that the risks of severe hypoglycaemia (defined as symptomatic hypoglycaemia requiring help from another person plus blood glucose < 2.5 mmol/l) were 1.3% and 3.3%, respectively. Unfortunately, the type of SU was not specified in the study [40]. A small study of 3 months’ duration, which included 29 patients receiving repaglinide alone and 27 receiving both repaglinide and metformin, reported no severe hypoglycaemic events [41].

Although rates of hypoglycaemia with SU are relatively low, particularly with the newer agents, around 636 000 patients with Type 2 diabetes in the UK receive SU, either alone or as part of combination therapy [42]. This equates, even at rates of 0.8% per annum for severe events requiring emergency assistance [22], to > 5000 patients experiencing a severe event each year.

### Insulin

Rates of hypoglycaemia with insulin vary according to the regimen and the stage of evolution of the person's diabetes. In the UKPDS, patients who were newly diagnosed at the start of the study and randomized to insulin therapy reported ‘any’ hypoglycaemia rates of around 33% at year 1 and around 43% at year 10 [21]. Corresponding rates for severe episodes (defined as episodes requiring third-party help or medical intervention) were approximately 1.2% and 2.2%, respectively.

Data derived from the DARTS-MEMO databases have revealed that 7.3% of patients with Type 2 diabetes treated with insulin suffered at least one episode of severe hypoglycaemia—a comparable figure to patients with Type 1 diabetes treated with insulin (7.1%) [22].

**FIGURE 2:**
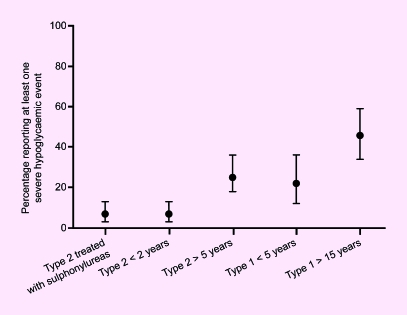
Proportion of patients with Type 2 and Type 1 diabetes of differing durations and receiving different regimens experiencing at least one severe hypoglycaemic attack during 9–12 months’ follow-up. All patients were receiving insulin except the group treated with sulphonylurea [35]. Reproduced from [35] with kind permission of Springer Science and Business Media.

One possible contributor to the difference in observed rate of severe hypoglycaemia between the patient populations of the UKPDS and DARTS-MEMO studies is duration of diabetes—patients were newly diagnosed at entry to UKPDS [21]. It is important also to look at the duration of insulin therapy when considering hypoglycaemia rates in patients with Type 2 diabetes treated with insulin. The Department for Transport study outlined above [35] found that 51% of patients taking insulin for < 2 years had experienced at least one hypoglycaemic episode during the 9–12 months of follow-up, compared with 64% of patients taking insulin for > 5 years. Rates of hypoglycaemia recorded by continuous glucose monitoring (defined as values of < 2.2 mmol/l for at least 20 min) were 20% and 22%, respectively. However, corresponding figures for severe hypoglycaemia were 7% and 25%. In comparison, the rate of severe hypoglycaemia in insulin-treated Type 1 patients of < 5 years’ duration was 22%, rising to 46% in patients with long duration (> 15 years) (Fig. 2).

The increase in severe episodes with duration of treatment has been confirmed by a UK-based retrospective study of 215 insulin-treated patients with Type 2 diabetes, which revealed that 15% had experienced severe hypoglycaemia in the preceding year (60 episodes in 32 people) [23]. In this study, 29 (13%) patients had been on insulin therapy for > 10 years, with an annual prevalence of severe hypoglycaemia of 31%.

However, insulin regimen is critical. To be included in the Department for Transport study [35], patients had to be on at least twice-daily insulin injections. Rates of symptomatic hypoglycaemia in Type 2 patients started on bedtime replacement of basal insulin using NPH insulin with metformin are approximately half those of patients using twice-daily insulin, despite larger insulin doses and lower HbA_1c_[43]. Severe hypoglycaemia did not occur in that study and was also absent from a later study of bedtime insulin and metformin using either NPH or a peakless insulin analogue [44]. Indeed, hypoglycaemia rates are no different in the early studies with bedtime NPH [43,45] and the trials of best start regimens with bedtime peakless analogue [46]. Hypoglycaemia rates are slightly higher when SUs are retained in the oral regimen, and best results seem to occur when these are replaced by using slightly higher starting doses of the basal insulin.

Despite these reassuring data, professional fear of hypoglycaemia often delays initiation of insulin therapy in Type 2 patients with suboptimal control on maximal oral therapy, to the detriment of glycaemic control and patient care [47].

### Incretin enhancement therapies: incretin analogues and dipeptidyl-peptidase 4 inhibitors

Newly emerging therapies based around enhancement of incretin action result in improved glycaemic control through a variety of mechanisms. The incretin, glucagon-like peptide (GLP)-1, released from the small intestine after eating, enhances insulin responses to glucose, as well as suppressing glucagon postprandially. It is too early to assess accurately the hypoglycaemic risk profile of such agents, although the glucose-lowering effect of GLP-1 appears to be glucose dependent [48].

Early clinical trials with the GLP-1 analogue exenatide, and the dipeptidyl-peptidase 4 inhibitors (such as sitagliptin and vildagliptin, which elevate endogenous GLP-1 concentrations by preventing its breakdown) do not seem to be associated with increased risk of hypoglycaemia when used as monotherapy [49] or in combination with insulin sensitizers (metformin and PPARγ-agonists) [50–54] in short-term clinical studies of between 24 and 52 weeks, although studies with exanatide have demonstrated increased risk of hypoglycaemia if used in association with SUs [55,56]. In a 30-week trial of exenatide vs. placebo in addition to treatment with SU [55], rates of mild/moderate hypoglycaemia were 3% (4/123) in the placebo group compared with 14% (18/125) in the exenatide 5 µg twice daily group, with no serious events in either arm. A further 30-week trial comparing exenatide and placebo and in addition to maximum dose combination therapy with SU and metformin, has revealed rates of mild/moderate hypoglycaemia of 13% (31/247) in the placebo group and 19% (47/245) in the exenatide 5 µg twice daily group [56].

The authors of both papers have suggested that the increased hypoglycaemia seen in the exanatide plus SU arms was a result of achieving lower levels of glycaemia coupled with the background hypoglycaemic risk associated with SU therapy [21,55,56].

The data with the incretin enhancement therapies thus suggests that the risk of hypoglycaemia is determined by the nature of the other glucose-lowering therapies used with them.

## The consequences of hypoglycaemia

### Clinical consequences

Mild symptomatic hypoglycaemia is not reported to have any serious clinical effects, apart from the potential for inducing defects in counterregulatory responses and impaired awareness to subsequent hypoglycaemia. Nevertheless, people with diabetes are fearful of hypoglycaemia (see below), and even clinically trivial events may be enough to inhibit concordance with therapy.

Severe hypoglycaemia is more serious, particularly in the elderly. In a prospective study of people aged > 80 years, with well-controlled (mean HbA_1c_ 5.1%) Type 2 diabetes, 25% of hospital admissions associated with diabetes were due to severe hypoglycaemia (defined as symptomatic event requiring third-party treatment and blood glucose < 2.8 mmol/l) [57]. Patients had considerable comorbidity (cardiovascular disease, dementia or diabetic complications).

In a retrospective cohort study of elderly patients (mean age 78 years) presenting to A&E departments in Tennessee with hypoglycaemia (defined as hospitalisation, A&E admission or death associated with hypoglycaemic symptoms and a blood glucose of < 2.8 mmol/l), almost all patients presented with neuroglycopenic symptoms and 49% presented with loss of consciousness. Approximately 5% were associated with stroke, myocardial infarction, transient ischaemic attack, injury or death, although the cause-and-effect relationship is not clear [19].

Severe hypoglycaemia is also associated with increased mortality. For example, in cases of severe hypoglycaemia induced by SU monotherapy, the overall mortality rate has been estimated as approximately 9%[58]. Deficits in cognitive functioning have been found to occur during hypoglycaemic events [8,9], and in one study over one-fifth of patients with Type 2 diabetes were found unconscious during a severe hypoglycaemic episode [29].

A retrospective review of 102 patients (90% with Type 2 diabetes) admitted to hospital with drug-induced hypoglycaemic coma revealed a mortality rate of 4.9% (*n* = 5) (all in patients with Type 2 diabetes) [16]. However, it was not possible to confirm whether the deaths were due to hypoglycaemia, since all five patients had serious comorbidities. Coma was associated with considerable morbidity, including head trauma, fracture, seizures, transient asymptomatic myocardial ischaemia and stroke.

In the UK, there are five fatal road traffic accidents each year and 45 serious events each month as a result of hypoglycaemia. Although the data do not differentiate between hypoglycaemia due to Type 1 or Type 2 diabetes, it is likely that a proportion of these incidents will involve patients with Type 2 diabetes [59].

Hypoglycaemia in people with Type 2 diabetes has a detrimental impact on HRQoL. This impact is apparent across different types of HRQoL measure. People who experienced hypoglycaemic symptoms had a lower mean health-related utility (HRU), as measured by the EQ-5D, compared with those who did not experience symptoms (0.70 vs. 0.77, *P* = 0.006) [60]. People with Type 2 diabetes have lower HRU as measured by EQ-5D than those with Type 1 diabetes [61]. Severity of the hypoglycaemia correlates with HRU, those with severe hypoglycaemia having the lowest HRU and HRQoL across all dimensions of the SF-36 except vitality [61]. The UKPDS has reported that Type 2 patients who experience more than two hypoglycaemic events during the study report more mood disturbance (problems with fatigue, tension, depression and anger) than those who do not report any events [62], and patients who experience these events report more generalized worries with their life and with their diabetes control than those not experiencing symptoms [60].

### Cost implications

The utilization of healthcare resources and treatment costs of hypoglycaemia in Type 2 diabetes varies according to country, and estimates will be affected by factors such as the prevalence of hypoglycaemia; classifications of hypoglycaemic events; patient characteristics, knowledge and attitudes to hypoglycaemic events; and varying quality and implementation of care across different healthcare systems.

Estimates vary for the amount of healthcare resources used by patients after a hypoglycaemic event, and these resources are almost certainly both underestimated and underutilized, as many patients will self treat an event and not mention the experience to their physician [29]. In one UK study, the mean numbers of primary care healthcare resources (visits to the nurse or physician) per patient with Type 2 diabetes in a 6-week period for mild/moderate and severe hypoglycaemia were reported as 11.5 and 13.2, respectively. To place this in a financial context, costs at these rates over a 6-week period for mild/moderate hypoglycaemia range from £287.50 if all consultations are with a GP to £92 if all consultations are with the Practice Nurse. Corresponding figures are £330 and £105.60 for severe hypoglycaemia [61,63]. However, a Canadian study has found that after mild/moderate and severe events, 84.5% and 83.1%, respectively, of the patients surveyed self-treated their hypoglycaemic event. Only 3.4% of those who reported a severe event requested an ambulance and only 5.5% had an A&E or hospital visit [29].

Scottish data based on documented resource use during 1 year show 7.3% of insulin-treated and 0.8% of SU-treated patients involved emergency services in a severe hypoglycaemic event, with > 80% of those events requiring an ambulance and 66% requiring Primary Care services or A&E. They estimated the annual direct cost of treating severe hypoglycaemia in excess of £13 million pounds for all patients with diabetes in the UK. Of the 244 episodes reported in the study, 57% were in patients with Type 2 diabetes. Therefore, we can estimate the cost of hypoglycaemia due to Type 2 diabetes at around £7.4 million [22]. The study included cost of ambulance, A&E departments and ward use at £127, £89 and £218 per day, respectively, but did not estimate the associated costs of any injury, disability or work loss associated with the acute event.

There is evidence to suggest that people with Type 2 diabetes lose on average 3 productive days (days lost from paid employment, non-paid normal activities and days requiring help with usual activities) following a severe hypoglycaemic attack [61]. In one study, over a 6-week period severe hypoglycaemia resulted in the loss of a mean of 8.6 productive days per patient with Type 2 diabetes [61].

Hospital admission due to hypoglycaemia in patients with Type 2 diabetes is associated with poor general condition after the event, concomitant illness and injuries sustained during the event [64]. A German study has estimated that costs for hypoglycaemia in Type 2 diabetes are substantially higher than in Type 1 patients, ($8000 compared with $44 300 dollars), reflecting longer length of hospital stay associated with older age, comorbidity and polypharmacy [64].

Data derived from the DARTS-MEMO databases reveal that 28% of episodes of severe hypoglycaemia (which were bad enough to merit involvement of emergency services) resulted in hospital admission [22]. The mean length of hospital stay was 4.4 days, which at a cost of £218 per day equates to £959 per hospitalisation for severe hypoglycaemia.

Other studies have reported the mean length of hospital stay associated with severe hypoglycaemia at between 6.6 and 9.5 days [64,65], with mean hypoglycaemic event duration reported between 12 and 72 h [16]. It should be noted that the rate of hospital admission may be affected by management protocols as well as individual medical need, as protocols for severe hypoglycaemia induced by SU therapy mandate admission to observe for recurrence of the hypoglycaemia, but such admissions, if uncomplicated, should be relatively short.

### Fear of hypoglycaemia and hypoglycaemia as a barrier to glycaemic control

Hypoglycaemia is clearly an important limiting factor in the glycaemic management of patients with diabetes and a significant barrier in terms of adherence to medication and achievement of the life-long goal to attain normoglycaemia [66]. Fear of hypoglycaemia is an additional psychological burden that patients with Type 2 diabetes experience [66]. A study that used the Hypoglycaemic Fear Survey, which combines a worry and behavioural scale [67], found that Type 2 diabetic patients reported increased fear of hypoglycaemia as the number of both mild/moderate and severe events increased. Women reported significantly more fear of hypoglycaemia than men [68].

It is suggested that the presence (or fear) of hypoglycaemia can limit the aggressiveness of drug therapy to achieve reduction of micro- and macrovascular complications, decrease adherence to diet and reduce patients’ willingness to take medications as directed [29,69]. The perceived risk of hypoglycaemia with insulin therapy has led to restrictions in licensing for driving.

Intensive therapy, designed to minimize the risk of long-term diabetic complications, has been associated with increased risk of severe hypoglycaemia, and fear of hypoglycaemia with a barrier to achieving and maintaining near-normoglycaemia. In one study, people with Type 2 diabetes with HbA_1c_ < 8.0% at baseline receiving a glibenclamide-containing regimen were up to 4.8 times more likely to experience hypoglycaemia than those with HbA_1c_ > 8.0%[70]. In the UKPDS [21] and a US Veterans Affairs study [71], those in the intensive therapy group reported significantly more hypoglycaemia than those on conventional therapy.

Hypoglycaemia has also been suggested as a private experience that is rarely discussed with healthcare providers [72], which may burden the patient more than physicians are aware. Sadly, patients reported to a UK survey that they received very little information and guidance from physicians regarding adverse effects of their glucose-lowering regimens. Only 5% of 165 patients with Type 2 diabetes were able to give the correct answers to questions regarding the adverse effects of SUs, and only 10% of people treated with a SU knew that it could cause hypoglycaemia [73].

Studies indicate that patients may balance their glucose levels against the risk of a hypoglycaemic event, with some choosing to keep their blood glucose higher than recommended to avoid hypoglycaemia [29]. Such patients may sacrifice the long-term benefits of reducing micro- and macrovascular complications [66]. With growing evidence that macrovascular disease may require lower mean glycaemia than that required for microvascular disease, the limitation of hypoglycaemia associated with current therapies may become more problematic, inhibiting patients’ ability to lower mean glucose concentrations sufficiently or long enough to impact on macrovascular end-points as required [74,75].

## Conclusions

Glucose-lowering therapies that are associated with hyperinsulinaemia that is not glucose dependent, such as the SUs and insulin, carry a risk for hypoglycaemia that is increased in older patients, those with longer duration of diabetes, lesser insulin reserve and other comorbidities, including renal impairment, hypothyroidism and defects of counterregulatory hormone secretion. The risk may be greater in the drive for intensified therapy and strict glycaemic control.

Severe hypoglycaemia also increases with increasing duration of Type 2 diabetes, and of insulin therapy, presumably as a result of increasing deficiency of endogenous, glucose-regulated insulin secretion, with rates of self-reported severe hypoglycaemia rising from 7 to 25% in those who have been taking insulin for > 5 years.

Although the rates of severe hypoglycaemia are lower in patients taking SUs, especially the newer third-generation agents, there are large numbers of patients taking these drugs in the UK, either alone or as part of combination therapy. Even at rates of 0.8% per annum for severe events requiring emergency care, this equates to > 5000 patients each year.

Hypoglycaemia has a substantial clinical impact in terms of mortality, morbidity and quality of life. The cost implications of severe episodes, both direct hospital costs and indirect costs due to inability to work, are considerable. Perhaps more important, hypoglycaemia and fear of hypoglycaemia limit the ability of current diabetes medications to achieve and sustain the degree of glycaemic control predicted to prevent the increased risk of diabetic complications.

Newer therapies which may carry a lower risk of hypoglycaemia, and more prudent use of older ones, may help patients achieve improved glucose control, defined in terms of both low HbA_1c_ and low risk of significant hypoglycaemia, for longer, and reduce the risk of diabetic complications.

## Competing interests

This work was supported by an unrestricted educational grant from Merck Sharp & Dohme Ltd. The views expressed in this publication are those of the authors, and not necessarily those of the publisher or sponsor. S.A.A. serves on advisory panels for Amylin Inc., Eli Lilly UK, Merck Sharp & Dohme, Novartis, Pfizer, and was an Association of British Clinical Diabetologists representative to NICE for the assessment of inhaled insulin. She did not receive any financial support for writing this paper. T.D. is a director of JB Medical Ltd, a medical education consultancy. JB Medical Ltd has been paid by Merck & Co, and its UK subsidiary Merck Sharp & Dohme Ltd, to produce marketing materials for sitagliptin and to write educational programmes and clinical papers in the area of diabetes. R.M. was on an internship sponsored by Merck & Co, Inc., NJ, USA. K.J. is an employee of Merck Sharp & Dohme (MSD) Ltd, the manufacturer of sitagliptin. K.J. also holds shares in the company.

R.M. carried out the initial Medline search. This was repeated by T.D. to ensure all relevant papers were identified, given the time lapse between R.M.'s original search and the writing of the paper. R.M. and T.D. provided S.A.A. with an overview of the Medline search and the key issues arising from their findings. S.A.A. and T.D. produced the initial manuscript. All other authors (R.M., K.J.) commented on the initial draft and T.D. and S.A.A. managed the incorporation of comments to produce a final draft manuscript for submission.

## Abbreviations

A&E: Accident and Emergency
ADA: American Diabetes Association
CDA: Canadian Diabetes Association
DARTS-MEMO: Diabetes Audit and Research in Tayside Scotland/Medicines Monitoring Unit Collaboration
EMEA: European Medicines Agency
GLP-1: glucagon-like peptide 1
GP: general practitioner
HRQoL: health-related quality of life
HRU: health-related utility
PPARγ: peroxisome proliferator-activated receptor gamma
SU: sulphonylurea
UKPDS: United Kingdom Prospective Diabetes Study
